# SIRT7: the seventh key to unlocking the mystery of aging

**DOI:** 10.1152/physrev.00044.2022

**Published:** 2023-09-07

**Authors:** Umar Raza, Xiaolong Tang, Zuojun Liu, Baohua Liu

**Affiliations:** ^1^Shenzhen Key Laboratory for Systemic Aging and Intervention (SKL-SAI), National Engineering Research Center for Biotechnology (Shenzhen), School of Basic Medical Sciences, Shenzhen University Medical School, Shenzhen, China; ^2^School of Biomedical Sciences, Hunan University, Changsha, China; ^3^School of Life Sciences, Hainan University, Haikou, China

**Keywords:** aging, SIRT7, stress response

## Abstract

Aging is a chronic yet natural physiological decline of the body. Throughout life, humans are continuously exposed to a variety of exogenous and endogenous stresses, which engender various counteractive responses at the cellular, tissue, organ, as well as organismal levels. The compromised cellular and tissue functions that occur because of genetic factors or prolonged stress (or even the stress response) may accelerate aging. Over the last two decades, the sirtuin (SIRT) family of lysine deacylases has emerged as a key regulator of longevity in a variety of organisms. SIRT7, the most recently identified member of the SIRTs, maintains physiological homeostasis and provides protection against aging by functioning as a watchdog of genomic integrity, a dynamic sensor and modulator of stresses. SIRT7 decline disrupts metabolic homeostasis, accelerates aging, and increases the risk of age-related pathologies including cardiovascular and neurodegenerative diseases, pulmonary and renal disorders, inflammatory diseases, and cancer, etc. Here, we present SIRT7 as the seventh key to unlock the mystery of aging, and its specific manipulation holds great potential to ensure healthiness and longevity.

## Graphical abstract

**Figure:**
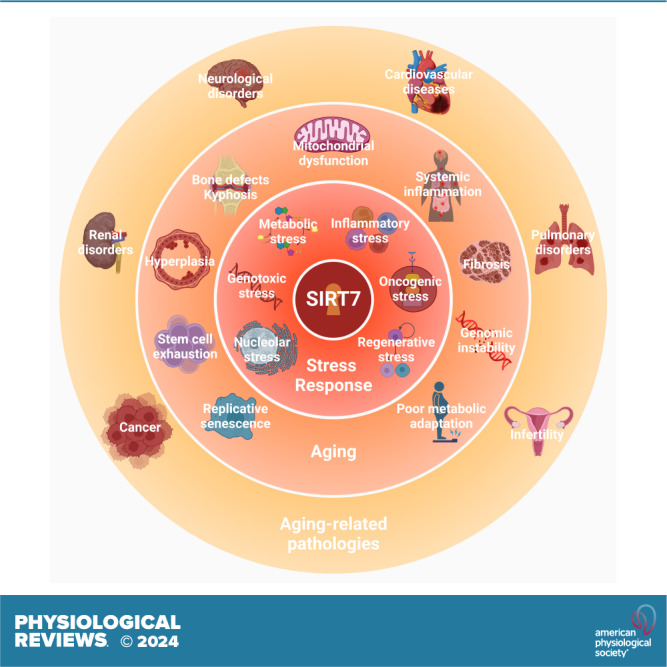

CLINICAL HIGHLIGHTS

- SIRT7 is a NAD^+^-dependent deacylase, mono-ADP-ribosyltransferase, and NAD^+^-independent RNA ac4C deacetylase.
- SIRT7 acts as an intrinsic defense against aging by orchestrating multiple stress responses.
- SIRT7 decline accelerates aging and makes one vulnerable to age-related pathologies.
- SIRT7 activators and gene manipulation hold potential as antiaging therapeutics.

## 1. INTRODUCTION

**FIGURE 1.**
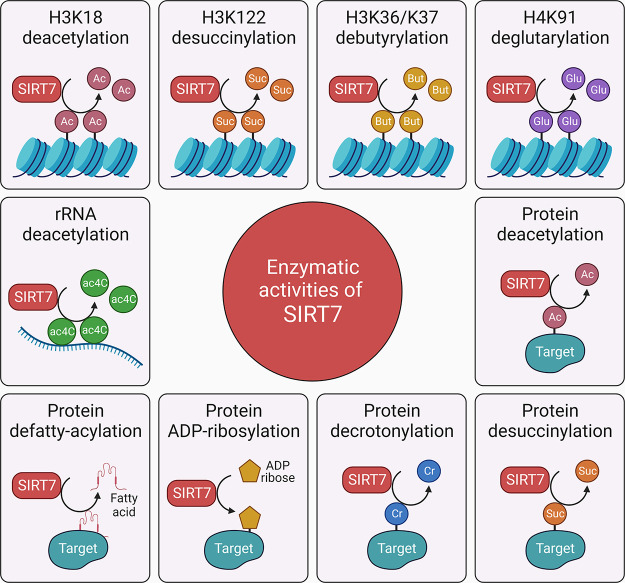
Enzymatic activities of SIRT7. SIRT7 functions as an epiregulator of the genome, transcriptome, and proteome through multiple enzymatic activities. It regulates gene expression through histone modifications including H3K18 deacetylation, H3K122 desuccinylation, H3K36/K37 debutyrylation, and H4K91 deglutarylation in a context-dependent manner. SIRT7 also deacetylates and desuccinylates nonhistone proteins. Additional SIRT7 activities identified to date include protein defatty-acylation, ADP-ribosylation, and decrotonylation, by which it contributes to maintain cellular homeostasis, and deacetylation of rRNA (ac4C) to regulate ribosome biogenesis.

Aging, or time-dependent physiological decline, is a chronic yet natural culmination of the loss of particular regenerative and bioprotective processes that occurs in the majority of living organisms (1). Characterized by a steady loss of physiological integrity, aging leads to impaired cellular and organ function as well as reduced metabolic adaptation. This in turn exposes humans to primary risk factors for pathologies such as diabetes, cardiovascular and autoimmune diseases, cancer, and neurodegenerative disorders, thereby increasing vulnerability to death (2, 3). Deciphering the mechanisms that underlie aging is invaluable in accomplishing the ultimate goal of improving human health. During the last three decades, aging research has advanced dramatically thanks to the findings on multiple evolutionarily conserved biochemical processes and genetic pathways that actively govern the rate of aging and longevity (4). In this context, the discovery of the nicotinamide adenine dinucleotide + (NAD^+^)-dependent protein deacetylase silent information regulator 2 (Sir2) at the end of the 1990s, and its identification as an important watchdog of longevity in yeast, prompted the scientific community to search for its mammalian homologs, particularly those found in humans (5, 6). There are now seven members of the sirtuin family (SIRT1–7), all of which are evolutionarily conserved and share an NAD^+^ binding domain. They were later classified as a SIRT-specific, nonclassical group (class III) of histone deacetylases (HDACs) or more accurately lysine deacetylases (KDACs) (7, 8). Further identification of subcellular localization and substrates (proteins and RNAs) has established SIRTs as key epiregulators of the genome, transcriptome, and proteome, which take part in and modulate a broad range of cellular and physiological processes, including genomic integrity, cell proliferation, cell cycle, apoptosis, metabolism, stress resistance, and aging (9, 10). SIRT7, the most recently identified SIRT member, is expressed from a single locus at chromosome 17q25.3 as a 10-exon transcript encoding a 400-amino acid protein with a molecular mass of almost 45 kDa (11). It is mainly localized to the nucleoli within the cell and is particularly abundant in the blood, bone marrow, liver, spleen, and testes and low in ovaries and skeletal muscle (9, 11, 12). The enzymatic activity of SIRT7 is primarily attributed to NAD^+^-dependent deacetylation (13), although desuccinylase (14), defatty-acylase (15), debutyrylase (16), deglutarylase (17), decrotonylase (18), mono-adenosine 5′-diphosphate (ADP)-ribosyltransferase (19), and NAD^+^-independent RNA deacetylase (ac4C) (20) activities have also been reported. The identification of numerous substrates for SIRT7 later established the enzyme as a key regulator of cell integrity in response to various endogenous and external stresses (21–23), affirming its role in cellular, tissue/organ, and organismal aging as well as age-related pathologies (FIGURE 1).

## 2. AGING DECLINES SIRT7 EXPRESSION AND ACTIVITY

**FIGURE 2.**
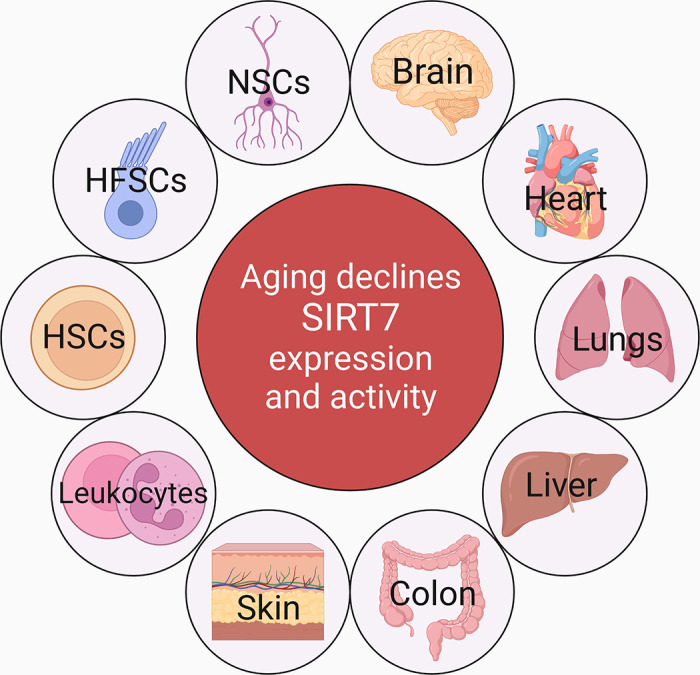
Aging declines SIRT7. Aging lowers SIRT7 expression and activity in multiple tissues and cell types including brain, heart, lungs, liver, colon, skin, leukocytes, and different stem cells. HFSCs, hair follicle stem cells; HSCs, hematopoietic stem cells; NSCs, neural stem cells.

Accumulating evidence from independent studies shows that the aging process disturbs both expression and activity of SIRT7. For instance, SIRT7 is lower in the colon tissues of 12-mo-old mice than in 2-mo-old animals (24). SIRT7 level is relatively constant in heart tissues during the first year of life in mice, but by the end of the second year it shows almost 40% reduction (25). Aged lungs also display lower SIRT7 in 18-mo-old mice compared with 5-wk-old mice (26). SIRT7 declines with age in murine hepatocytes, correlating with increased rDNA methylation (27), and in rat liver tissues (28, 29), which promotes p53-dependent cellular senescence (28). An age-related decline in SIRT7 is observed in leukocytes (30) and hematopoietic stem cells (HSCs) (31). Moreover, the levels of histone acetylation are higher in skin tissue of elderly than young individuals, attributable to decreased expression of HDACs including SIRT7 (32). With age, SIRT7 level decreases in hair follicle stem cells (HFSCs), and this loss delays hair maturation and growth (33). SIRT7 expression declines in hippocampal neural stem cells (NSCs) over time, which increases mitochondrial protein folding stress with age (34). In addition, the level of NAD^+^, a cosubstrate for multiple enzymes including SIRTs, declines with aging, thereby limiting global SIRT7 activity regardless of its expression (35, 36). Together, SIRT7 level and activity are primarily declined in multiple tissues with aging and hold potential to be targeted at the clinical level to ensure longevity (FIGURE 2).

## 3. SIRT7: AN INTRINSIC DEFENSE AGAINST LIFETIME STRESSES

During our lifetime, cells in our body are constantly exposed to various external and endogenous stresses that induce short-term and/or persistent damages. Multiple signaling pathways have been evolved to counteract these stresses, referred to as adaptive stress response, thus maintaining tissue homeostasis. The balance between stress-induced damage and adaptive stress response is therefore crucial in determining the pace of aging (37). Multiple stress signals including nucleolar, genotoxic, metabolic, inflammatory, oncogenic, and regenerative stresses are sensed through SIRT7, which works to limit the impact and duration of damage by coordinating optimal adaptive response via regulating downstream signaling cascades.

### 3.1. Nucleolar Stress Response

The nucleolus is the cellular factory for ribosome biogenesis. It is formed around the ribosomal DNA (rDNA), where a complex assembly of RNA polymerase (RNA pol), rRNA, and small nucleolar RNA (snoRNA) along with multiple regulatory, processing, and maturation factors governs production of ribosomal proteins and subsequent export to cytoplasm (38). Owing to its repetitive nature, high transcription rates, and clustering of replication origins and replication fork pause sites, rDNA is susceptible to double-strand breaks (DSBs) and repeat instability. This introduces morphological and functional alteration in the nucleolus over time and drives senescence (39). Primarily localized to the nucleolus, SIRT7 not only ensures rDNA stability but also governs ribosome biogenesis at multiple levels from rDNA transcription to rRNA maturation (22). SIRT7 recruits DNA methyltransferase DNMT1, the nucleolar remodeling complex (NoRC), and another sirtuin member, SIRT1, to rDNA repeats to promote chromatin silencing and condensation (40, 41). It also promotes RNA polymerase I (RNA Pol I) recruitment to rDNA by interacting with upstream binding factor 1 (UBF1) (42) and by deacetylating nuclear protein fibrillarin (FBL) (43) and RNA Pol I subunit PAF53 (44), thereby enhancing rDNA transcription. SIRT7 deacetylates DExD-box RNA helicase (DDX21), thereby resolving R loops formed between DNA and nascent RNA during transcription. If R loops are not fixed, RNA Pol stalls and DSBs are introduced (45). During mitosis, SIRT7 is inactivated through cyclin-dependent kinase 1 (CDK1)-cyclin B-governed phosphorylation and activated through a dephosphorylation-dependent conformational rearrangement in telophase, which ensures onset of rDNA transcription as the cell exits from mitosis (42). Conversely, SIRT7 maintains ribosomal homeostasis by epigenetically repressing the expression of ribosomal protein S7 (RPS7), a ribosomal protein involved in the DNA damage response (DDR) and cell death (46). At rRNA maturation stage, SIRT7 promotes U3 snoRNA-dependent cleavage and generation of 18S rRNA by deacetylating U3-55k and enhancing its binding to U3 snoRNA (47). All these findings pinpoint a central role for nucleolar SIRT7 in maintaining the global protein pool by regulating rDNA integrity and ribosome biogenesis. SIRT7 also regulates RNA Pol II-driven transcription of small nuclear RNAs (snRNAs) and mRNAs by deacetylating CDK9, which activates the elongation factor P-TEFb and RNA Pol III-driven transcription of various transfer RNAs (tRNAs) (48). This is achieved through interaction with general transcription factor IIIC polypeptide 1 (GTF3C1) of the transcription factor TFIIIC2 complex (49); thus SIRT7 contributes to the maintenance of cellular homeostasis at yet another level.

**FIGURE 3.**
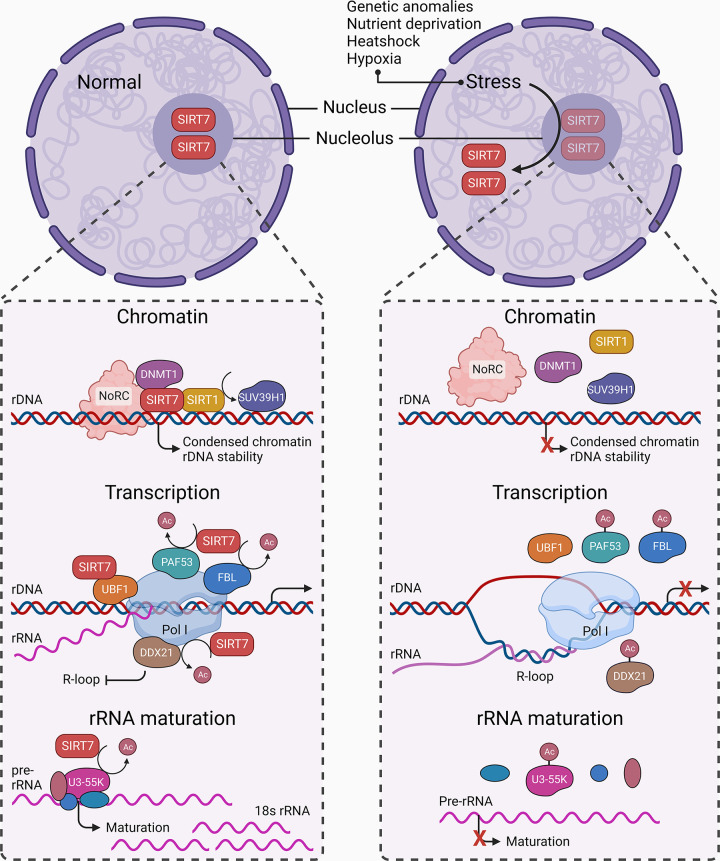
SIRT7 and nucleolar stress response. Under steady state (*left*), SIRT7 resides in the nucleolus, where it recruits DNA methyltransferase 1 (DNMT1), nucleolar remodeling complex (NoRC), and SIRT1 to rDNA locus and subsequently suppressor of variegation 3–9 homolog 1 (SUV39H1). These factors keep chromatin in a condensed state and maintain rDNA integrity. During transcription, SIRT7 interacts with upstream binding factor 1 (UBF1), deacetylates fibrillarin (FBL) and RNA Pol I subunit PAF53 to facilitate rDNA transcription, and deacetylates DExD-box RNA helicase 21 (DDX21) at transcription machinery to inhibit R-loop formation. During rRNA maturation, SIRT7 deacetylates U3-55k, which facilitates U3 small nucleolar RNA (snoRNA)-dependent cleavage and generation of 18S rRNA. Stress signals such as genetic anomalies, nutrient deprivation, heat shock, and hypoxia induce ribosomal or nucleolar stress (*right*) that leads to SIRT7 exclusion from the nucleolus. This compromises rDNA integrity and inhibits rDNA transcription and rRNA maturation. Arrowhead, activation; blunt head, inhibition; circle head, relation; bright, active axes; dim: underactive axes.

Intracellular and extracellular stimuli such as genetic anomalies in ribosomal proteins, ribosomal binding factor (RBF) deficiency, nutrient starvation, hypoxia, and heat shock induce nucleolar or ribosomal stress response. This response, depending upon the severity of stresses, often results in metabolic reprogramming, cell cycle arrest, senescence, or apoptotic cell death (50, 51). Under stress conditions, the release of SIRT7 from the nucleolus leads to reduced RNA Pol I recruitment to rDNA and rapid transcriptional repression due to the hyperacetylation of PAF53 and FBL in the transcription complex (43, 44). Nutrient starvation leads to SIRT7 exclusion from the nucleolus to conserve energy by inhibiting rDNA transcription (52). Likewise, pharmacological inhibition of SIRT7 activity by sirtinol inhibits the onset of rDNA transcription in late mitosis (42). In addition, stress-dependent translocation of SIRT7 to extranucleolar space leads to the hyperacetylation of U3-55k protein and defects in rRNA maturation and accumulation of intermediary transcripts (47). The energy-dependent nucleolar silencing complex (e-NOSC) senses nutrient deprivation and thus maintains compact heterochromatin at rDNA repeats via suppressor of variegation 3–9 homolog 1 (SUV39H1)-dependent histone methylation and SIRT1-mediated histone deacetylation (53). Of note, SUV39H1 is recruited to rDNA sites in a SIRT1-dependent manner (53); but SIRT1 itself relies on SIRT7 for recruitment to rDNA (41) (FIGURE 3).

**FIGURE 4.**
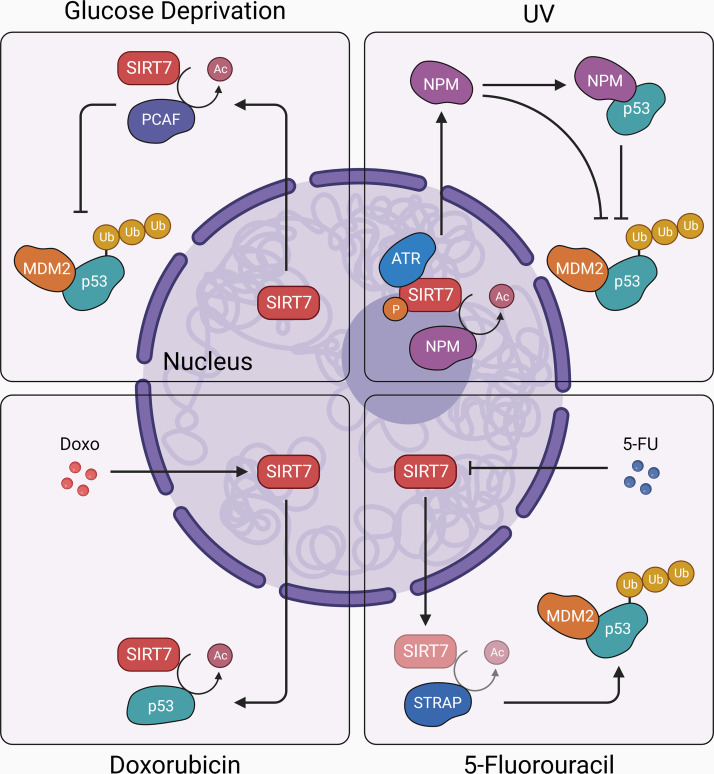
p53 stabilization downstream of SIRT7. SIRT7 regulates p53, a master regulator of cellular stress response, through multiple mechanisms. Upon glucose deprivation, SIRT7 exits from the nucleolus and deacetylates p300/CBP-associated factor (PCAF), which sequesters and induces MDM2 degradation, thereby promoting p53 stabilization (*top left*). Ultraviolet light (UV) promotes p53 stabilization downstream of the SIRT7-nucleophosmin (NPM) axis (*top right*). Treatment with chemotherapeutic agent doxorubicin (Doxo) promotes SIRT7 exclusion from the nucleolus, which directly interacts with p53 and stabilizes it through deacetylation (*bottom left*). On the other hand, treatment with chemotherapeutic agent 5-fluorouracil (5-FU) inhibits SIRT7-mediated deacetylation of serine-threonine kinase receptor-associated protein (STRAP) to stabilize p53 (*bottom right*). Arrowhead, activation; blunt head, inhibition; bright, active axes; dim, underactive axes.

Stabilization of tumor suppressor p53 is a key event in the cellular stress response in general and is a particular marker of nucleolar stress response. As such, robust physiological p53 activity is necessary for healthy aging and preservation of tissues from aging-related damage. Alternatively, excessive p53 activation compromises healthiness through widespread apoptosis, whereas loss or reduction of p53 activity leads to oncogenic transformation (54). Notably, SIRT7 tightly regulates p53 activity in multiple ways. SIRT7 deacetylates acetyltransferase p300/CBP-associated factor (PCAF) at lysine 720 (K720), which then sequesters MDM2 and promotes its degradation, thus promoting p53 stabilization and subsequent cell cycle arrest (55). Alternatively, SIRT7-mediated deacetylation directs the release of nucleophosmin (NPM) from the nucleolus to the nucleoplasm, where it binds to MDM2 and prevents p53 degradation (56). NPM may also directly interact with and stabilize p53 (57). In addition, SIRT7 released into extranucleolar space can directly acetylate p53 and favor cell survival by suppressing proapoptotic function of p53 (58), although contradictory findings that question whether p53 is a direct target of SIRT7 (9, 13) warrant further exploration (FIGURE 4).

### 3.2. Genotoxic Stress Response

Time-dependent accumulation of DNA damage because of nucleotide misincorporation, defective DNA replication, mutations in DNA repair enzymes, or exposure to exogenous genotoxins drives aging (59, 60). The resultant genomic instability negatively affects cell and tissue homeostasis by inducing gene expression changes, cellular senescence or apoptosis, or functional decline of tissues/organs. Thus genomic instability is potentially the unifying cause of aging at the root of other hallmarks of aging (61). Of various DNA damages, DSBs pose the greatest threat to genomic integrity, upon which cells initiate DNA damage response (DDR) to repair the lesions either by homologous recombination (HR) or nonhomologous end joining (NHEJ) (21). Although the role of SIRT7 in HR is reported (62), our current understanding of the exact molecular mechanisms involved is limited at best. On the other hand, the role of SIRT7 in orchestrating NHEJ repair is well documented. Upon DSBs, activated ataxia-telangiectasia mutated (ATM) phosphorylates histone H2AX to generate γ-H2AX, which, through a series of mediatory modifications in histones at damage sites, guides the recruitment of 53BP1 as an effector of the NHEJ pathway (63, 64). SIRT7 is recruited to DSB sites in a poly(ADP-ribose) polymerase (PARP)-dependent manner and promotes deacetylation of H3K18, which favors the recruitment of 53BP1 and NHEJ repair (65). In turn, SIRT7 hyperactivates PARP1 through the ADP ribosylation process, promoting PARP-mediated recruitment of other DDR-associated molecules to DNA damage sites (19). In addition, SIRT7 facilitates chromatin condensation by catalyzing histone H3K122 desuccinylation at DSB sites, and thus promotes DNA repair and cell survival (14). The accumulation of succinate and succinyl-CoA upon succinyl dehydrogenase (SDH) depletion promotes chromatin succinylation, which recapitulates SIRT7 deficiency-related defects in the DDR and thus sensitizes cells to genotoxic stress (66). Moreover, SIRT7 catalyzes H4K91 deglutarylation to ensure chromatin compaction during DNA repair (17). At the end of the repair, SIRT7 is again recruited to DNA damage sites, where it deacetylates activated ATM and tags it for dephosphorylation by p53-induced phosphatase 1 (WIP1) and then dimerization into an inactive form (67). SIRT7 also coordinates with acetyl transferase hMOF to regulate acetylation and deacetylation of p62; acetylated p62 is translocated to nucleus, recruits APE1 to the error site, and promotes base-excision repair (BER) and cell survival (68).

**FIGURE 5.**
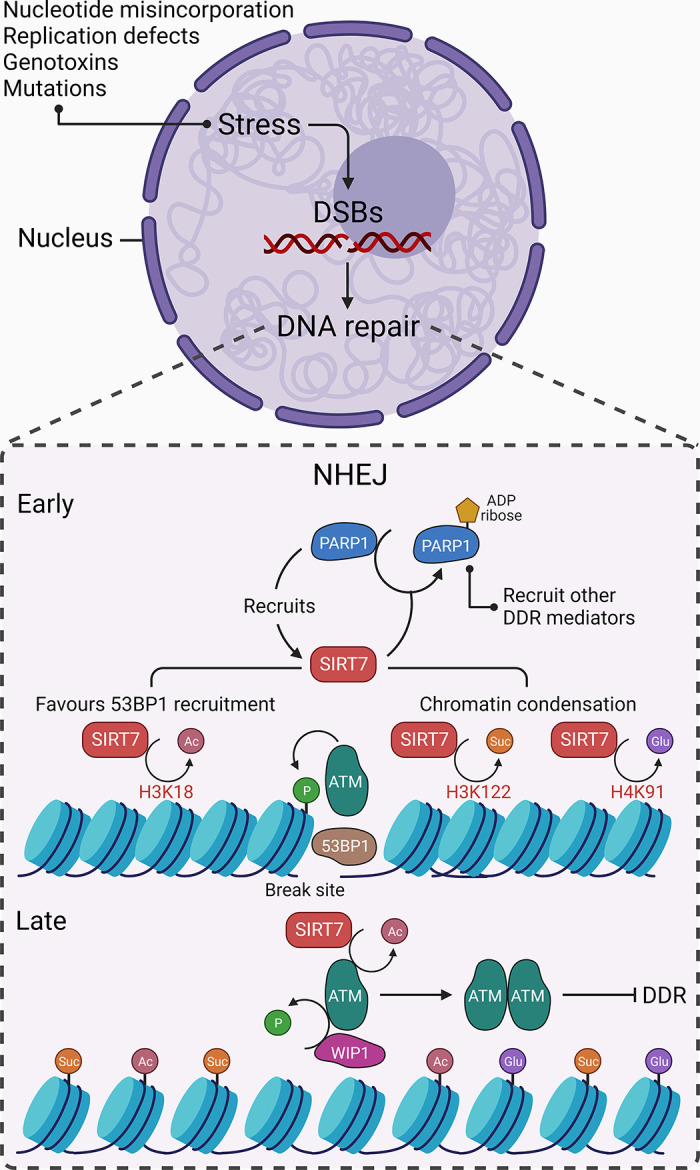
SIRT7 and genotoxic stress response. Nucleotide misincorporation, replication defects, exposure to genotoxins, and mutations introduce genotoxic stress via double-strand breaks (DSBs), upon which cells initiate DNA damage response (DDR) to repair the damage. During nonhomologous end joining (NHEJ)-mediated DNA repair, SIRT7 is recruited to the DNA damage site in a poly(ADP-ribose) polymerase (PARP)1-dependent manner, which in turn, promotes ADP-ribosylation of PARP1 to facilitate the recruitment of other DDR mediators. Once recruited to DSBs, SIRT7 favors 53BP1 recruitment by deacetylating H3K18 and promotes chromatin condensation by H3K122 desuccinylation and H4K91 deglutarylation. Toward the end of repair, SIRT7 deacetylates ataxia-telangiectasia mutated (ATM) that facilitates p53-induced phosphatase 1 (WIP1)-mediated dephosphorylation and dimerization of ATM into an inactive form. Arrowhead, activation; blunt head, inhibition; circle head, relation.

Most DDR mediators converge at p53, which not only orchestrates the downstream damage repair mechanisms but also decides cell fate, i.e., cell cycle arrest, senescence, or apoptosis (69). Under ultraviolet (UV)-induced genotoxic stress, another key DDR element named Ataxia telangiectasia and Rad3 related (ATR) phosphorylates SIRT7 and enhances its enzymatic activity. In turn, SIRT7 deacetylates NPM, which upon release into the nucleoplasm targets MDM2, resulting in rapid p53 accumulation and enhanced p53-dependent cell cycle arrest to ensure genomic integrity (56). The treatment of cells with the DNA damaging agent 5-fluorouracil (5-FU) leads to SIRT7 degradation and subsequent hyperacetylation of serine-threonine kinase receptor-associated protein (STRAP), which stabilizes p53 by preventing ubiquitination-mediated degradation (70). As such, the elevated SIRT7 confers resistance to chemotherapeutic agent doxorubicin-induced apoptosis by delaying the p53 response and attenuating the stress-activated kinases JNK and p38 MAPK, thereby shifting cellular fate toward senescence (71). Thus, the stress-induced, SIRT7-mediated p53 response is a complex process that depends upon the nature and intensity of the stress. Overall, pleiotropic roles of SIRT7 in orchestrating genotoxic stress response make it indispensable for maintaining genomic integrity and thus antagonizing the aging process (FIGURE 5).

### 3.3. Metabolic Stress Response

Nutrient imbalance due to either insufficient or excess nutrient intake puts metabolic strain on cells, leading to excessive production of reactive oxygen species (ROS) and causing damage to cellular components and cell death. Cellular NAD^+^ is actively consumed during energy production through glycolysis; therefore, the dependence of SIRTs on NAD^+^ for most enzymatic activity directly links them to cellular metabolic status and energy conservation in response to metabolic stress (72, 73). Upon glucose starvation, adenosine 5′-monophosphate (AMP)-activated protein kinase (AMPK), the cellular monitor of energy, phosphorylates SIRT7 and promotes its release from the nucleolus; such exclusion of SIRT7 causes hyperacetylation of the RNA Pol I subunit PAF53 and halts the process of rDNA transcription and preserves energy (44, 52). SIRT7 senses glucose availability through its methylation at arginine 388 (R388), mediated by protein arginine methyltransferase 6 (PRMT6). The resultant decrease in SIRT7 H3K18 deacetylase activity epigenetically promotes mitochondria biogenesis and cellular respiration, whereas AMPK abrogates the PRMT6-SIRT7 interaction upon glucose deprivation (74). SIRT7 can directly modulate glycolysis by deacetylating, thus inhibiting the activity of phosphoglycerate kinase 1 (PGK1) (75). Alternatively, during glucose deprivation SIRT7 promotes transcription of the glucose-6-phosphatase catalytic subunit (G6PC) through the ETS transcription factor (ELK4) to regulate gluconeogenesis. This process is fine-tuned by ubiquitin-specific peptidase 7 (USP7), which, under normal conditions, negatively regulates SIRT7 deacetylase activity, but such interaction is attenuated during glucose starvation (76). In another scenario, day-to-night light cues and restricted feeding elevate body temperature, inducing transcription of heat shock protein 70 (HSP70) in the liver. HSP70, in turn, promotes proteasomal degradation of SIRT7 in liver tissues, the latter of which deacetylates and tags cryptochrome circadian regulator 1 (CRY1) for F-box and leucine rich repeat protein 3 (FBXL3)-mediated ubiquitination and degradation. CRY1 interacts with glucocorticoid receptors and inhibits gluconeogenesis (77). Hypoxia-inducible factors (HIFs; HIF1 and HIF2) attenuate glucose oxidation in the tricarboxylic acid (TCA) cycle by upregulating pyruvate dehydrogenase kinase 1 (PDK1) expression, which, in turn, inactivates the rate-limiting enzyme pyruvate dehydrogenase (PDH) (78). SIRT7 fine-tunes this process by downregulating transcriptional activity of HIFs (79). Auto-ADP-ribosylated SIRT7 is recognized by and interacts with the ADP-ribose binding protein mH2A1, which regulates the localization of SIRT7 to certain intergenic regions involved in orchestrating calorie restriction (CR)-specific response. In particular, this SIRT7/mH2A1 axis regulates the expression of genes involved in cAMP signaling and autophagy in response to glucose starvation in vitro and to CR in mouse liver tissues in vivo (80). These findings suggest a pivotal role for SIRT7 in glucose metabolism that also has implications in glucose starvation and antiaging nutritional interventions like CR and fasting.

Aging-related changes in metabolism of lipids and their metabolites put enormous strain on the body in the form of lipotoxicity and associated diseases (81). Studies in genetically modified mice have deepened our understanding of SIRT7’s role in lipid metabolism during aging. *Sirt7*-deficient mice (which have exons 4–11 replaced with the LacZ gene) experience chronic hepatosteatosis like human fatty liver disease. Because of lower secretion of very-low-density lipoprotein (VLDL), *Sirt7*-knockout (KO) mice accumulate higher levels of triglycerides in the liver than wild types. Restoring SIRT7 in the liver normalizes triglyceride levels in these mice. Mechanistically, SIRT7 interacts with the Myc transcription factor to silence ribosomal protein transcription and alleviate endoplasmic reticulum (ER) stress to reverse fatty liver disease. Liver-specific SIRT7 overexpression protects mice with diet-induced obesity from developing ER stress-induced fatty liver (82). An alternate *Sirt7*-knockout mouse model (with exon 6–9 deletion) has high triglyceride levels and high free fatty acid accumulation in plasma, and increased blood lactate levels. The incidence of hepatosteatosis in these mice is attributed to mitochondrial dysfunction. Mechanistically, a lack of SIRT7-mediated deacetylation of the GA-binding protein transcription factor subunit beta 1 (GABPβ1) obstructs mitochondrial gene transcription, resulting in impaired mitochondrial homeostasis (83). In line with this finding, the phenotype of liver-specific lysine-specific demethylase-1 (LSD1)-knockout mice mirrors GABPβ1 hyperacetylation due to reduced NAD^+^ synthesis and NAD^+^-dependent SIRT7 deacetylase activity. Despite obvious mitochondrial dysfunction, these mice are protected against hepatosteatosis (84), plausibly because of incomplete loss of SIRT7 activity or because of SIRT7-independent roles of LSD1.

**FIGURE 6.**
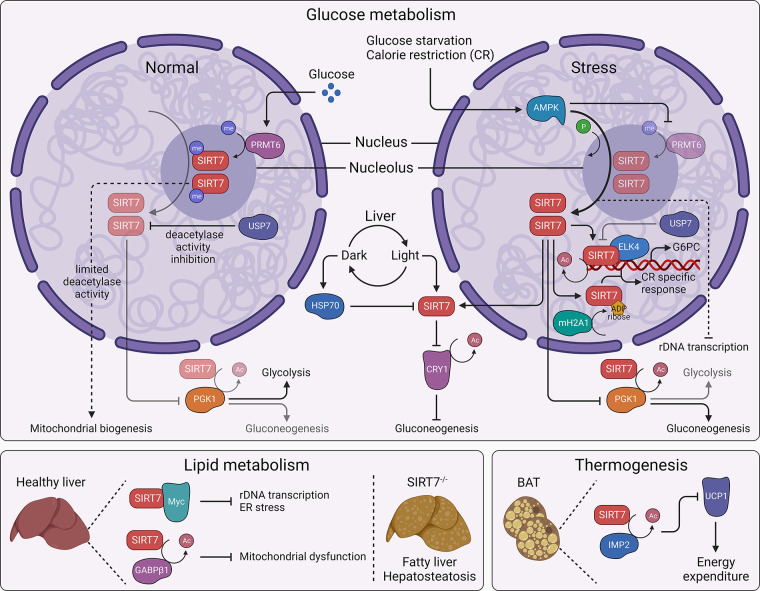
SIRT7 and metabolic stress response. SIRT7 senses glucose imbalance (*top*). Under steady state (*left*), high glucose activates protein argenine methyltransferase 6 (PRMT6), which methylates and limits SIRT7 deacetylase activity, thus promoting mitochondrial biogenesis. Low glucose (*top right*) upon glucose starvation or calorie restriction (CR) activates AMP-activated protein kinase (AMPK), which phosphorylates SIRT7, leading to its exclusion from the nucleolus, limiting rDNA transcription to conserve energy. During daytime, SIRT7 regulates gluconeogenesis in the liver by deacetylating cryptochrome circadian regulator 1 (CRY1), whereas heat shock protein 70 (HSP70) reduces SIRT7 level during dark. SIRT7 deacetylates and inhibits phosphoglycerate kinase (PGK1), thereby shifting glycolysis to gluconeogenesis under stress. In addition, glucose starvation limits ubiquitin specific peptidase 7 (UPS7)-mediated SIRT7 inactivation, upon which SIRT7 interacts with ETS transcription factor (ELK4) to transcribe the glucose-6-phosphatase catalytic subunit (G6PC) and promote gluconeogenesis and mH2A1-driven ADP-ribosylation of SIRT7 orchestrates CR-specific response. SIRT7 regulates lipid metabolism (*bottom left*). In the liver, SIRT7 interacts with Myc to control rDNA transcription and limit endoplasmic reticulum (ER) stress and deacetylates GA binding protein transcription factor subunit beta 1 (GABPβ1) to inhibit mitochondrial dysfunction, thereby maintaining proper lipid metabolism. *Sirt7*^−/−^ mice experience hepatosteatosis and fatty liver disease. SIRT7 also preserves energy during thermogenesis (*bottom right*). In brown adipose tissue (BAT), SIRT7 deacetylates insulin-like growth factor 2 mRNA-binding protein 2 (IMP2), which inhibits uncoupling protein 1 (UCP1) and controls energy expenditure on thermogenesis. Arrowhead, activation; blunt head, inhibition; bright, active axes; dim, underactive axes.

Nutrients are metabolized into energy that is utilized to fuel varying physiological processes. Brown adipose tissue (BAT) regulates thermogenesis through energy expenditure in an uncoupling 1 (UCP1)-dependent manner, but elderly individuals may struggle to maintain body temperature because of age-related loss of BAT and thermogenesis. Recently, SIRT7 has been shown to control energy expenditure in BAT. Mechanistically, it works as an energy saving factor by deacetylating insulin-like growth factor 2 mRNA-binding protein 2 (IMP2), which, in turn, inhibits the translation of UCP1 leading to minimized energy expenditure on thermogenesis (85) (FIGURE 6).

### 3.4. Inflammatory Stress Response

**FIGURE 7.**
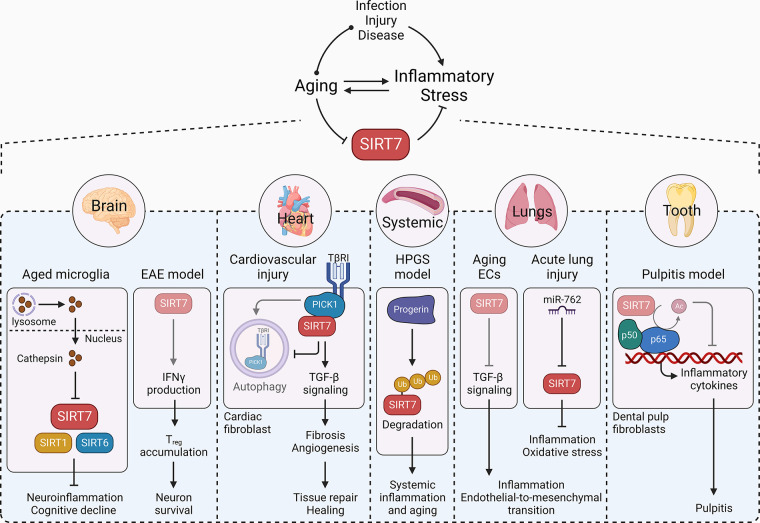
SIRT7 and inflammatory stress response. Tissue insults due to infections, injuries, and diseases over time introduce inflammatory stress. SIRT7 is intertwined with inflammatory stress, provides protection against local (tissue specific) and systemic inflammation, and promotes tissue repair and healing. In brain, SIRT7, along with SIRT6 and SIRT1, inhibits neuroinflammation and cognitive decline, whereas lysosomal rupture-released cathepsin suppresses SIRT activity in the aged microglia. In the experimental autoimmune encephalomyelitis (EAE) model of neuroinflammation, the downregulation of SIRT7 limits interferon-gamma (IFN-γ) production, regulatory T cell (T_reg_) accumulation, and neuron survival. Upon cardiovascular injury, SIRT7 interacts with protein kinase-Cα (PICK1) and limits PICK1-driven autophagic degradation of TGF-β receptor I (TβR1) in cardiac fibroblasts. This, in turn, promotes angiogenesis, tissue repair, and healing. In a mouse model of Hutchinson–Gilford progeria syndrome (HPGS), progerin promotes proteasome-dependent SIRT7 destabilization, which enhances systemic inflammation and aging. In lung endothelial cells (ECs), SIRT7 inhibits TGF-β signaling to limit inflammation and endothelial-to-mesenchymal transition, while aging-related decline in SIRT7 compromises the axis. In an acute lung injury model, miR-762 inhibits *SIRT7* to promote inflammation and oxidative stress. In dental pulp fibroblasts, loss of *SIRT7* causes p65 hyperacetylation and thus inhibits transcription of inflammatory cytokines and causes inflammatory pulpitis. Arrowhead, activation; blunt head, inhibition; circle head, relation; bright, active axes; dim, underactive axes.

The tissue insults caused by infection, injury, or disease introduce immunogenic and inflammatory responses that promote healthy healing and tissue repair. However, the effects of dysregulated and prolonged inflammatory responses accumulate with aging and lead to disease pathogenesis (86). An inflammo-protective role of SIRT7 first became evident from the finding that *Sirt7^−^*^/−^ mice develop inflammatory cardiomyopathy (25). The mutant animals have reduced angiogenesis and a lower inflammatory response upon myocardial infarction and hindlimb ischemia, evidenced by impaired blood flow recovery, decreased expression of angiogenic factors, and accumulation of immune cells. At the molecular level, SIRT7 promotes transforming growth factor-beta (TGF-β) signaling by interacting with protein kinase-Cα (PICK1) and limiting PICK1-driven autophagic degradation of TGF-β receptor I (TβRI), which triggers subsequent angiogenic and inflammatory response in cardiac tissue. Together, these effects lead to quicker healing (87). In a mouse model of *Lmna*^G609G^ mutation (progerin) knockin, a causal mutation of Hutchinson–Gilford progeria syndrome (HGPS), dysfunction of vascular endothelial cells induces an inflammatory response owing to proteasome-dependent SIRT7 destabilization. The endothelial cell-specific restoration of SIRT7 ameliorates inflammatory stress and aging-related features and extends the life span (88). A decline in SIRT7 activity exacerbates acute lung injury, arising from direct (pneumonia) or indirect (sepsis or trauma) insults, by inducing an inflammatory environment and promoting fibrosis during aging. SIRT7 silencing induces endothelial-to-mesenchymal transition in lung vascular endothelial cells by promoting TGF-β signaling, which compromises the endothelial barrier and increases vascular permeability and inflammatory responses, leading to lung fibrosis (26). In an intratracheally instilled LPS-based in vivo acute lung injury model, SIRT7 is transcriptionally repressed by upregulation of microRNA (miR)-762, whereas SIRT7 overexpression has a pulmonary-protective effect, including inhibition of inflammation and reduced oxidative stress (89). Microglia are the primary immune cells of the nervous system. Aging-related loss of lysosomal membrane integrity leads to leakage of cathepsin B, which may accumulate in the nucleus of these senescent microglia and degrade SIRT7, SIRT6, and SIRT1, thus leading to neuroinflammation and cognitive decline during aging (90). In experimental autoimmune encephalomyelitis (EAE; a mouse model of autoimmune-driven neuroinflammation), *Sirt7* deficiency causes inflammatory stress because of diminished peripheral interferon-gamma (IFN-γ) production and failed accumulation of regulatory T cells in the central nervous system. This decreases the survival of newly generated neurons in the hippocampus (91). Moreover, decreased SIRT7 level in dental pulp fibroblasts hyperactivates NF-κB signaling, which leads to pulpitis, an inflammatory dental disease. Restoring SIRT7 level alleviates inflammatory response both in vitro and in vivo (92), although the direct interaction between SIRT7 and NF-κB components remains elusive under this specific scenario. Overall, SIRT7 serves as an important element in immune response against inflammatory stresses that has far-reaching impacts on aging (FIGURE 7).

### 3.5. Oncogenic Stress Response

**FIGURE 8.**
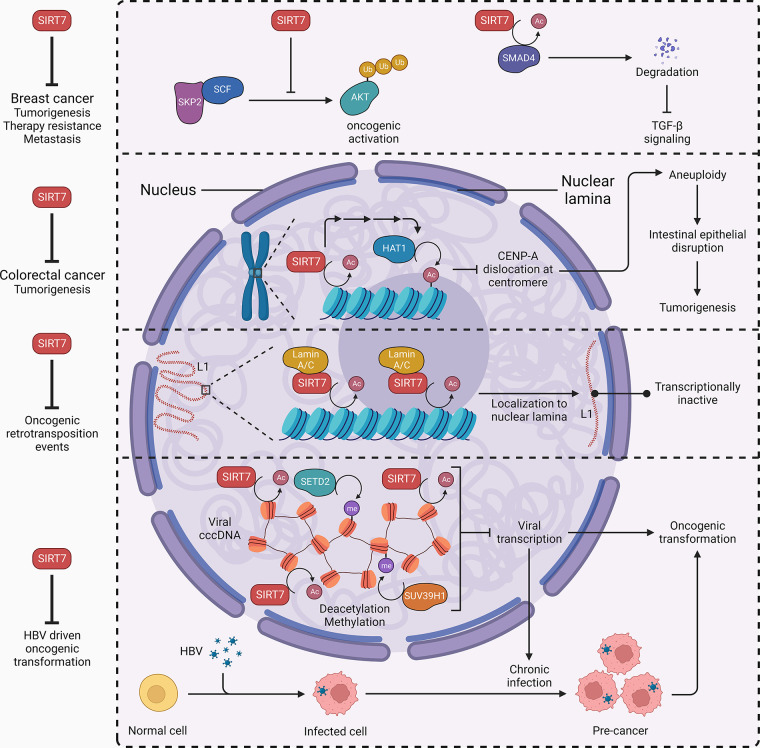
SIRT7 and oncogenic stress response. SIRT7 inhibits S-phase kinase-associated protein 2-Culin1-F-box (SKP2-SCF)-mediated oncogenic activation of protein kinase B (AKT) and deacetylates SMAD family member 4 (SMAD4) to limit transforming growth factor β (TGF-β) signaling. These events suppress breast cancer tumorigenesis, therapy resistance, and metastasis. SIRT7 maintains centromere integrity and inhibits colorectal cancer tumorigenesis. SIRT7 deacetylates H3K18, which promotes histone acetyletransferase 1 (HAT1)-mediated histone deacetylation. It inhibits centromere protein A (CENPA) dislocation at centromere and promotes intestinal epithelial integrity. SIRT7 inhibits retrotransposition events by Lamin A/C-assisted chromatin deacetylation that localizes L1 transposons to nuclear lamina and renders them transcriptionally inactive. SIRT7 limits hepatitis B virus (HBV) transcription to inhibit virus-driven oncogenic transformation. It deacetylates histones in viral covalently closed circular DNA (cccDNA) along with SET domain containing 2 (SETD2) and suppressor of variegation 3–9 homolog 1 (SUV39H1)-driven methylation to silence viral genome transactivation. Arrowhead, activation; blunt head, inhibition; circle head, relation.

Although the role of SIRT7 in cancer progression has been extensively studied (discussed in detail in sect. 5.6), few studies have explored the effect of oncogenic stress on SIRT7. The polyoma middle T antigen (PyMT) oncoprotein activates the Ras and the phosphatidylinositol 3-kinase (PI3K)/protein kinase B (AKT) oncogenic pathways to induce the formation of mammary tumors (93). In mouse mammary tumor virus (MMTV)-driven PyMT transgenic mice, loss of one allele of *Sirt7* promotes the initiation and progression of breast cancer and metastasis to the lung (94). In addition, the same *Sirt7* insufficiency induces much larger and more numerous papillomas in mice upon sequential treatment with 7,12-dimethylbenz (alpha) anthracene (DMBA) and 12-*O*-tetradecanoyl-phorbol-13-acetate (TPA) (94, 95). *Sirt7* overexpression suppresses S-phase kinase-associated protein 2 (Skp2)-Culin1-F-box (SCF) E3 ligase-mediated AKT ubiquitination and TGF-β/SMAD family member 4 (SMAD4) signaling, thus antagonizing the growth of breast tumors and metastasis to distal organs (94, 96). The mutation in the adenomatous polyposis coli (APC) gene predisposes animals to multiple intestinal neoplasia (Min). *Sirt7* deficiency impedes histone acetyltransferase 1 (HAT1) activity and leads to hypoacetylation of histone H4, which dislocates histone variant centromere protein A (CENPA) from centromeres and compromises nucleosome assembly (24). In *Sirt7*^−/−^; *Apc*^Min/+^ mice, increased Wnt signaling, dysregulated chromosome segregation, and impaired DNA repair drive an early tumor incidence and increase tumor load compared with *Apc*^Min/+^ mice (24). Long interspersed element-1 (LINE-1 or L1) is a retrotransposon that over time introduces mutations through self-propagation in the genome. Somatic cells evolve multiple mechanisms to restrict L1 activity, but age-related dysfunction in these processes may lead to cancer onset and progression. SIRT7 epigenetically represses the transcription of L1 elements through H3K18 deacetylation, which with the help of Lamin A/C promotes the recruitment of L1 elements to the nuclear lamina and protects cells from oncogenic retrotransposition events (97). Hepatitis B virus (HBV) infection predisposes humans to liver cancer. After infection, the covalently closed circular DNA (cccDNA) of HBV resides in host-cell nucleus in the form of minichromosomes. The desuccinylase activity of SIRT7 modulates chromatin structure in conjunction with the histone methyltransferase SUV39H1 and SET domain containing 2 (SETD2), impeding viral transcription and oncogenic transformation (98). Overall, SIRT7 plays different oncoprotective roles in a tissue-specific and stress-dependent manner (FIGURE 8).

### 3.6. Regenerative Stress Response

The self-renewal and differentiation capacity of stem cells makes them invaluable for proper growth and healthy aging, as they actively facilitate tissue regeneration. A decline in the stem cell pool or loss of self-renewal and differentiation abilities contributes to faster aging (99). HSCs are multipotent precursors of blood lineages that remain in a quiescent state unless required to differentiate. SIRT7 interacts with nuclear respiratory factor 1 (NRF1) to inhibit the downstream transcription of mitochondrial ribosomal proteins (mRPs), an event that is involved in mitochondrial biogenesis during HSC differentiation. SIRT7 progressively declines in HSCs during aging, and loss of *Sirt7* in mice compromises hematopoiesis (31). Of note, reduced *SIRT7* expression is associated with hematopoietic disorders like acute myeloid leukemia (AML) and chronic myeloid leukemia (CML) (30). *SIRT7* expression is also positively correlated with mobilization of HSCs from bone marrow to peripheral blood in patients with lymphoproliferative disorders (100). SIRT7 level gradually decreases in hair follicular stem cells (HFSCs) with aging, and loss of *Sirt7* in mice delays hair follicle cycling and hair growth by obstructing telogen-to-anagen transition. Mechanistically, SIRT7 deacetylates the nuclear factor of activated T cells 1 (NFATc1), suppresses its nuclear retention, and thus promotes PA28γ-dependent proteasomal degradation, thereby abrogating quiescence of HFSCs (33). SIRT7 is also downregulated during senescence of human mesenchymal stem cells (MSCs). It prevents MSCs from senescence by complexing with nuclear lamina and heterochromatin proteins, thus maintaining a repressed state of heterochromatin at the nuclear periphery. On the other hand, *SIRT7* deficiency results in a loss of heterochromatin, derepression of LINE1, and activation of innate immune signaling via the cyclic GMP-AMP synthase (cGAS)-stimulator of interferon genes (STING) pathway, thereby accelerating MSC senescence (101).

Osteoblasts, which are derived from bone marrow mesenchymal stem cells (BMSCs), differentiate to bone-forming osteocytes, whereas osteoclasts originated from a hematopoietic lineage regulate the removal of old bone matter. Osteoblast differentiation is carried out by osteogenic transcription factors such as runt-related transcription factor 2 (RUNX2) and zinc finger transcription factor SP7/Osterix (OSX) (102). Osteoblast-specific *Sirt7* deletion hinders bone formation as it enhances OSX transactivation activity through deacylation at K368, which also promotes SIRT1-dependent depropionylation of OSX (103). miR-193a inhibits BMSC osteogenic differentiation and induces osteoporosis by targeting *SIRT7* and promoting NF-κB signaling. Plasma samples and bone tissues from patients with osteoporosis contain elevated miR-193a but a decreased SIRT7 level. Treatment of BMSCs with the SIRT activator resveratrol promotes osteogenic differentiation and thus alleviates osteoporosis (104). On the other front, osteoarthritis is an age-related progressive degradation of articular cartilage, which causes severe inflammation and pain in the joints and disability. SIRT7 level is reduced in degenerated cartilage of osteoarthritis patients and is insufficient to orchestrate the autophagy response that is necessary to protect chondrocytes from degeneration (105). A better mechanistic understanding of SIRT7 imbalance will be of clinical importance in developing therapies against osteoarthritis.

Adipocytes, which can be derived from MSCs, form adipose tissue that stores energy in the form of lipids. SIRT7 controls adipose tissue homeostasis and fat accumulation by restricting SIRT1 activity, which upon autodeacetylation blocks peroxisome proliferator-activated receptor (PPAR)-γ2 and adipocyte differentiation. Elevated SIRT1 activity diminishes lipogenesis and deposition in *Sirt7*-KO mice (106). SIRT7 also regulates adipose tissue homeostasis by directly interacting with PPAR-γ2, namely deacetylating PPAR-γ2 at K382, which impairs PPAR-γ2 transcriptional activity, leading to dysregulation of lipogenic genes (107). miR-93 keeps the self-renewal capacity of adipocyte precursor in check by targeting T-Box transcription factor 3 (*TBX3*) and controls the differentiation of adipocytes by targeting *SIRT7* (108).

**FIGURE 9.**
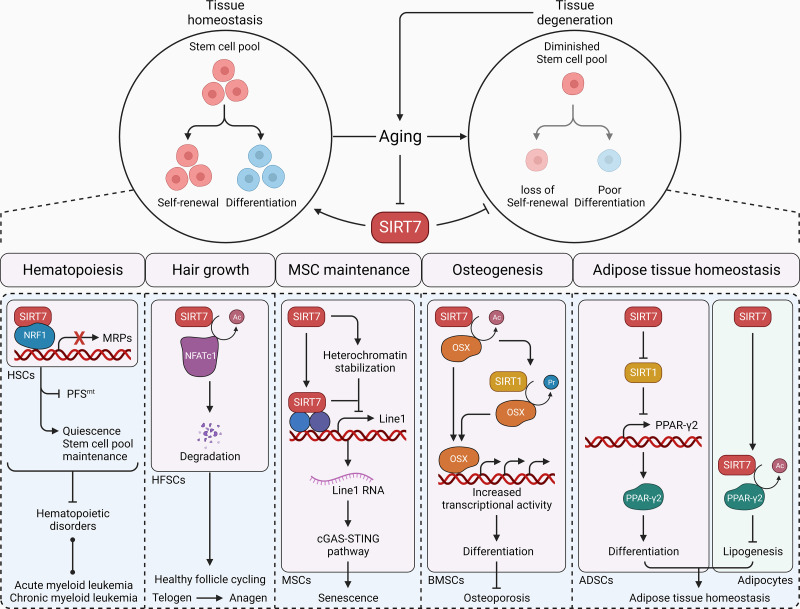
SIRT7 and regenerative stress response. Stem cell pool and self-renewal and differentiation abilities diminish along with aging. SIRT7 decline in expression and activity accelerates stem cell aging. In hematopoietic stem cells (HSCs), SIRT7 interacts with NRF1 and inhibits transcription of mitochondrial ribosomal proteins (MRPs), thereby limiting mitochondrial protein folding stress and differentiation of hematopoietic stem cells (HSCs). This aids in stem cell pool maintenance and provides protection against hematopoietic disorders such as acute and chronic myeloid leukemia. In hair follicle stem cells (HFSCs), SIRT7 deacetylates and tags nuclear factor of activated T cells 1 (NFATc1) for degradation that results in healthy follicle cycling between telogen and anagen stage during aging. SIRT7 stabilizes heterochromatin state and inhibits transcription of L1 transposons to protect mesenchymal stem cells (MSCs) from senescence. In bone marrow MSCs (BMSCs), SIRT7 deacetylates and promotes SIRT1-dependent depropionylation of osteogenic transcription factor Osterix (OSX) that enhances osteogenic differentiation and inhibits osteoporosis. In adipose-derived stem cells (ADSCs), SIRT7 inhibits SIRT1 histone deacetylase activity to promote transcription of peroxisome proliferator-activated receptor (PPAR)-γ2 and subsequent ADSC differentiation. Alternatively, SIRT7 can directly interact and deacetylate PPAR-γ2 to limit lipogenesis in mature adipocytes, thereby maintaining adipose tissue homeostasis. Arrowhead, activation; blunt head, inhibition; bright, active axes; dim, underactive axes; cGAS-STING, cyclic GMP-AMP synthase-stimulator of interferon genes.

Surges in maternal glucocorticoid levels during late gestation may cause defects in hippocampal neural stem cells that lead to cognitive decline and mood disorders in children. In an experimental model, treatment of pregnant mice with dexamethasone (a synthetic glucocorticoid) inhibits neural stem cell proliferation in embryos by suppressing *SIRT7* expression while promoting p21-mediated cell cycle arrest (109). In addition, the neuroprotective and antiapoptotic effects of ferulic acid in neural stem cells are mediated by the upregulation of SIRT7 as well as SIRT1 and MDM2 (110). Likewise, increased miR-152 and decreased posttranscriptional target *SIRT7* induces replicative senescence in dental pulp stem cells (111). Collectively, SIRT7 counteracts regenerative stress and aging by tightly regulating tissue regeneration (FIGURE 9).

## 4. EXPERIMENTAL PERTURBATIONS OF SIRT7 ACCELERATE AGING

Over the last decade, multiple groups have developed *Sirt7*-KO animals with different strategies and have substantially improved our knowledge about the role of SIRT7 in systemic aging and longevity. *Sirt7*-KO mice experience increased lipid deposition, attributed to uncontrolled lipogenesis in liver, along with reduced secretion of very-low-density lipoprotein (VLDL), thereby resulting in chronic hepatosteatosis resembling human fatty liver disease (82). Intriguingly, although hepatosteatosis is generally associated with obesity in humans, *Sirt7*-KO mice are leaner than littermate control mice (82); this is likely attributable to diminished lipogenesis in adipose tissues (106). Similarly, high-fat diet (HFD) does not induce obesity in *Sirt7*-KO mice, which rather have decreased epididymal white adipose tissue (WAT) compared with control mice. Moreover, liver-specific *Sirt7*-KO mice developed WAT normally and gained body weight similar to control mice (112). General reconstitution of SIRT7 to physiological levels reversed fatty liver phenotype, and liver-specific SIRT7 overexpression relieved fatty liver disease in diet-induced obese mice (82).

Multisystemic mitochondrial dysfunction is observed in an alternate *Sirt7*-KO mouse model. In line with the abovementioned findings, this model experiences hepatic microvesicular steatosis along with elevated levels of plasma triglycerides and free fatty acids (83). These mutant mice also exhibited elevated lactate levels following a glucose challenge and a 30-min treadmill run, accompanied by decreased endurance performance, which is often seen in mitochondrial disease. The exercise intolerance was observed in *Sirt7*-KO mice, which is thought to be primarily due to cardiac issues (83) as SIRT7 is expressed minimally or not at all in skeletal muscle (11, 83). Increased mitochondrial protein folding stress in activated hippocampal NSCs is acquired during aging, which leads to neural hyperactivity, cognitive decline, and neurodegenerative disorders. *Sirt7*-KO mice exhibit neural hyperactivity, memory deficit, and cognitive decline early in life (34, 113), whereas overexpression of SIRT7 in aged mice improves neurogenesis and cognitive functions by alleviating mitochondrial protein folding stress (34). Energy expenditure through thermogenesis declines with age, plausibly because of loss of BAT. Whole body *Sirt7*-KO mice harbor less interscapular BAT compared with control mice, and these mice as well as BAT-specific *Sirt7*-KO mice exhibit higher body temperature due to uncontrolled energy expenditure through thermogenesis (85, 112), thereby suggesting pivotal roles of SIRT7 in energy homeostasis during aging.

*Sirt7*-KO mice experience heart hypertrophy due to cardiac cell inflammation and fibrosis, leading to inflammatory cardiomyopathy (25), and are prone to cardiac rupture following heart attack (87). Cardiomyocyte-specific *Sirt7*-deficient mice exhibit increased cardiac fibrosis and reduced cardiac contractile function (114). Consistently, an alternate *Sirt7*-KO mouse line developed multisystemic mitochondrial dysfunction and cardiac dysfunction (83). At a young age, hearing in *Sirt7*-KO mice is comparable to that in control animals. However, these mice exhibit hearing loss as they age (83). HSCs in *Sirt7*-KO mice also experience mitochondrial stress-related functional decline similar to that observed with aging, characterized by increased apoptosis, loss of quiescence, low reconstitution capacity, and myeloid-biased differentiation (31). Moreover, depletion of *Sirt7* induces intestinal anomalies recapitulating age-related intestinal dysfunction. This is associated with increased levels of senescence markers p16^Ink4a^, p21^Cip1^, and p53 in colon (24). *Sirt7*-KO animals also exhibit other aging-related changes including kyphosis (excessive curvature of upper back generally observed in the elderly) (25), although they are resistant to aging- and forced exercise-induced osteoarthritis (115). As females age, fertility tends to decline. Fertility span is further reduced in *Sirt7*-KO female mice, resulting in fewer litters produced and fewer pups born in each litter compared with wild-type females (116), suggesting accelerated aging.

**FIGURE 10.**
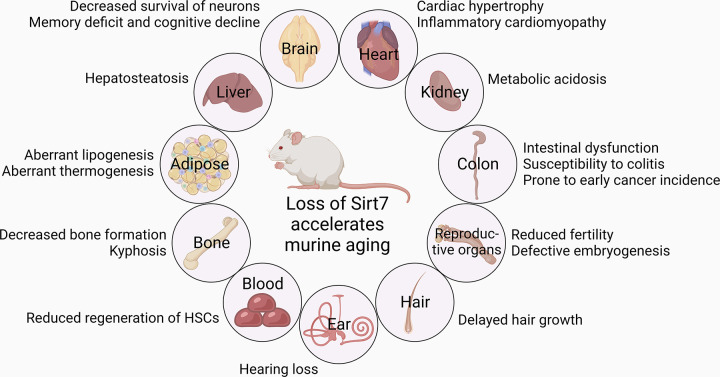
SIRT7 and aging. *Sirt7* deficiency accelerates murine aging with a disease-associated shorter life span. Defects and disorders observed in brain, heart, liver, kidney, adipose, colon, bone, reproductive organs, blood, hair, and ears of *Sirt7*^−/−^ mice are shown. HSCs, hematopoietic stem cells.

Although inheritable mutations of *SIRTs* have not been identified in human genetic disorders to date, they are closely related to premature aging disease, namely Hutchinson–Gilford progeria syndrome (HGPS), caused by aberrant splicing of the *LMNA* gene (117). Lamin A interacts with and thus activates SIRT1 deacetylase; compromised SIRT1 deacetylase activity caused by mutated Lamin A (progerin) leads to early depletion of MSCs and accelerates aging in a mouse model (118). Lamin A also enhances the deacetylase and mono-ADP-ribosyltransferase activity of SIRT6 to safeguard genome integrity (119). Lamin A prevents SIRT7 from proteasomal degradation in mouse vascular endothelium, whereas progerin loses such function, thereby triggering systemic aging. Additionally, recombinant adeno-associated virus serotype 1 (rAAV1)-mediated SIRT7 gene delivery-targeted vascular endothelium rejuvenates aging tissues and extends life span (88) (FIGURE 10).

## 5. SIRT7 PROTECTS AGAINST AGING-RELATED PATHOLOGIES

Age-related physiological decline makes the body prone to diseases over time. Diseases contribute to and accentuate the aging process. Extensive studies over the years have proved the involvement of SIRT7 in aging-related pathologies. In this section, we summarize the roles that SIRT7 plays in regulating major human pathologies.

### 5.1. Cardiovascular Diseases

**FIGURE 11.**
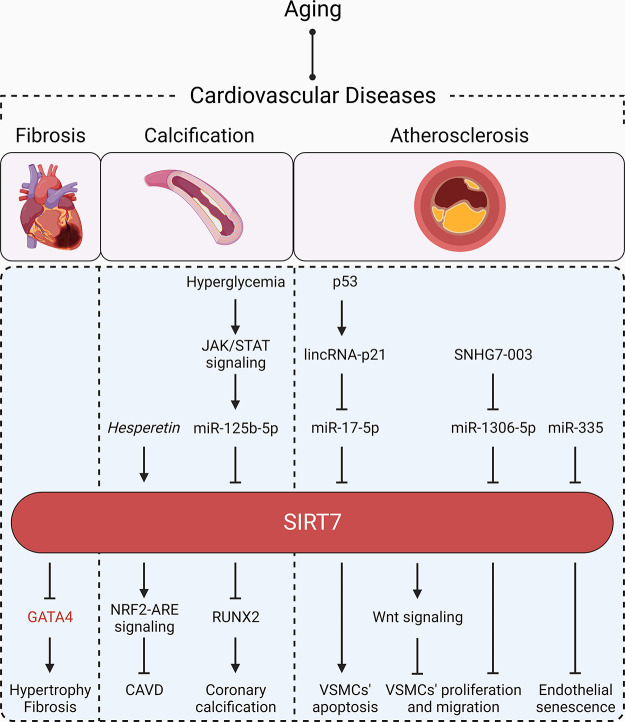
SIRT7 and cardiovascular diseases. Aging is associated with cardiovascular diseases. SIRT7 counteracts cardiac fibrosis, calcification, and atherosclerosis through different molecular mechanisms. The upstream regulators and downstream effectors of SIRT7 activity in these cardiovascular diseases are presented. SIRT7 suppresses hypertrophy and fibrosis by directly inhibiting GATA binding protein 4 (GATA4). SIRT7 limits calcification by upregulating NRF2-ARE signaling and inhibiting runt-related transcription factor 2 (RUNX2) expression. SIRT7 prevents atherosclerosis by fine-tuning vascular smooth muscle cell (VSMC) proliferation, migration, and apoptosis and by inhibiting endothelial senescence. SIRT7 and its direct targets are shown in red. Therapeutic interventions are written in italics. Arrowhead, activation; blunt head, inhibition; circle head, relation. CAVD, calcific aortic valve disease.

Although activation of cardiac fibroblasts and differentiation into myofibroblasts is a physiological process in response to injury or stress, myofibroblast persistence causes cardiac fibrosis. Cardiac fibrosis is characterized by excessive deposition of extracellular matrix (ECM) and may lead to cardiac dysfunction and subsequent heart failure (120). *Sirt7*-KO mice experience heart hypertrophy due to cardiac cell inflammation and fibrosis, which results in inflammatory cardiomyopathy. In addition, increased basal level of apoptosis in primary cardiomyocytes renders *Sirt7*-KO mice susceptible to oxidative and genotoxic stress (25). Cardiomyocyte-specific *Sirt7*-deficient mice exhibit increased cardiac fibrosis and reduced cardiac contractile functions. In response to pressure overload, SIRT7 is upregulated in myocardial tissue and mediates antihypertrophic and antifibrotic effects by deacetylating and thus regulating GATA binding protein 4 (GATA4) transcriptional activity (114). Persistent fibrosis along with progressive calcification of aortic valve leaflets eventually obstructs left ventricular outflow and induces aortic stenosis, leading to calcific aortic valve disease (CAVD). Treatment with the dietary supplement hesperetin protects the aortic valve interstitial cells (VICs) against CAVD-associated valve damage. Mechanistically, hesperetin directly binds to and upregulates SIRT7, which, in turn, activates nuclear factor erythroid 2-related factor 2 (NRF2)-antioxidant response elements (ARE) signaling and inhibits oxidative stress, mitochondrial dysfunction, inflammatory response, and apoptosis in VICs (121). SIRT7 deacetylates and inhibits osteogenic transcription factor RUNX2 in myeloid cells, thereby repressing coronary artery calcification over time. Hyperglycemia upregulates expression of miR-125b-5p mediated by Janus kinase (JAK)/signal transducer and activator of transcription (STAT) signaling, which inhibits *SIRT7* and promotes myeloid cell-driven coronary calcification in diabetic individuals (122) (FIGURE 11).

Atherosclerosis is characterized by the accumulation of plaques in arteries, which narrows the path for circulating blood. Vascular smooth muscle cells (VSMCs) reside in the medial layer of the trilayered (intima, media, adventitia) vessels. Despite their active roles in neointimal formation upon vascular injury, VSMCs may dedifferentiate and/or undergo phenotypic switching to multiple cell types, including ECM-producing fibrous cap cells, macrophage-like cells, and foam cells, which together are involved in plaque formation in the blood vessels (123). SIRT7 limits the unnecessary proliferation of VSMCs and provides protection against atherosclerosis in multiple ways. One such mechanism involves p53-dependent lincRNA-p21 expression that downregulates miR-17-5p in VSMCs, and the latter upregulates *SIRT7*. This, in turn, promotes apoptotic cell death in VSMCs, contributing to the protection against atherosclerosis progression. Notably, p53, lincRNA-p21, and SIRT7 are downregulated in the peripheral blood of patients with atherosclerosis, whereas the level of miR-17-5p is upregulated (124). In the oxidized low-density lipoprotein (ox-LDL)-induced model of atherosclerosis, SIRT7 inhibits proliferation and migration of human VSMCs by enhancing Wnt/β-catenin signaling (125). In a similar in vitro model, miR-1306-5p aids the progression of atherosclerosis by downregulating *SIRT7*, thus promoting proliferation, migration, and invasion of VSMCs. As an approach to counteract atherosclerosis, upregulation of the long noncoding RNA (lncRNA) SNHG7-003, which is downregulated in response to ox-LDL treatment of VSMCs, sequesters miR-1306-5p from *SIRT7* transcripts, thus preventing its downregulation (126). In addition, *SIRT7* is targeted by miR-335 to induce endothelial senescence and contributes to the pathogenesis of atherosclerosis (127). In summary, these studies show that SIRT7 plays a protective role against the development and progression of atherosclerosis by tightly controlling proliferation, migration, and survival of VSMCs (FIGURE 11).

### 5.2. Renal Disorders

Hyperglycemia-induced apoptosis of podocytes in kidneys contributes to pathogenesis of diabetic nephropathy. SIRT7 plays a protective role under these conditions; the upregulation of miR-20b in podocytes targets *SIRT7* to promote apoptosis during hyperglycemia (128). Metabolic acidosis, characterized by excessively acidic body fluids, induces *SIRT7* expression in kidney cells, which, in turn, stabilizes K^+^-Cl^−^ cotransporter (KCC4) through deacetylation. In response to ammonium chloride challenge, *Sirt7*-KO mice have lower expression of KCC4 and higher metabolic acidosis than wild-type animals (129). Hyperglycemic stress can persist even after glucose normalization, namely hyperglycemic memory, which may aid in developing diabetic nephropathy. In glomerular endothelial cells, ETS transcription factor (ELK1) translocates to nucleus and thus promotes death-associated protein kinase 3 (DAPK3) transcription, which augments NF-κB-mediated glomerular inflammation and renal dysfunction even after glucose normalization. Notably, SIRT7 and ELK1 mutually inhibit each other at the DAPK3 promoter; thus, forced SIRT7 overexpression may kill the hyperglycemic memory of the ELK1-DAPK3 axis and alleviate diabetic nephropathy (130). In addition, hypertension can lead to renal injury and dysfunction characterized by partial epithelial-to-mesenchymal transition (EMT), extensive lipid peroxidation, fibrosis, and ferroptosis. SIRT7 overexpression mitigates this hypertension-related structural and functional damage in kidney cells by hyperactivating the Kruppel-like factor 15 (KLF15)/NRF2 axis in an angiotensin II (ANG II) infusion-induced hypertensive mouse model (131). These findings suggest that SIRT7 protects against renal damage.

### 5.3. Pulmonary Disorders

Pulmonary fibrosis is a severe life-threatening condition, attributed to aging-related mechanisms. All SIRTs are downregulated in primary lung fibroblasts from patients with idiopathic pulmonary fibrosis (IPF) or systemic sclerosis-associated interstitial lung disease (SSc-ILD) compared with those from healthy control subjects. SIRT7 was reduced the most between disease and control individuals and inhibits lung fibrosis by limiting TGF-β/SMAD3 signaling-mediated expression of alpha smooth muscle actin (α-SMA) and collagen (132). An alternate fibrosis-causing mechanism exists downstream of TGF-β via the activation of glutaminase 1 (GLS1). TGF-β-induced inhibition of SIRT7 promotes the acetylation of forkhead box (FOX)O4 transcription factor, and, once acetylated, FOXO4 cannot repress GLS1 expression. Meanwhile, SMAD2/3 increases GLS1 expression, resulting in fibrosis (133). SIRT7 level is decreased in a mouse model of lung fibrosis induced by bleomycin, and pharmacological inhibition of GLS1 reverses the fibrosis (132, 133).

### 5.4. Neurodegenerative Disorders

Parkinson’s disease (PD) is characterized by the loss of dopaminergic neurons in the central nervous system. In an animal model that phenocopies PD, an age-dependent decline in SIRT7 was observed in different brain regions including cerebellum, brain stem, cerebral cortex, and basal ganglia (134). In a cellular model of PD, chemical-induced oxidative insult downregulates SIRT levels (including SIRT7) in an autophagy-dependent manner (135). A recent study suggests that F-box-only protein 7 (FBXO7) also promotes the ubiquitination and proteasomal degradation of SIRT7 upon chemical-induced oxidative insult, thereby suppressing cytoprotective effects of SIRT7 and contributing to the pathogenesis of PD (136). These studies suggest that SIRT7 has neuroprotective effects and an age-dependent decline in SIRT7 expression may make brain cells susceptible to oxidative damage-induced PD.

### 5.5. Infertility

In females, oocyte maturation through meiosis provides the basis of sexual reproduction. Meiosis in oocytes starts before birth but halts at the dictyate stage of prophase I until ovulation occurs. During ovulation, a small portion of oocytes resume meiosis and complete the maturation process to release the egg (137). In vitro, SIRT7 inhibition affects oocyte quality by disrupting cytoskeletal organization during meiosis and inducing oxidative stress. In line with this, SIRT7 overexpression improves fertility in obese female mice by relieving oxidative stress and meiotic defects in oocytes (138). A more detailed study in *Sirt7*-KO female mice showed that age-related decline in fertility is due to increased oocyte apoptosis and a diminished oocyte pool. Here, meiotic progression is delayed, attributable to DNA damage caused by defective chromosome synapsis that leads to meiotic arrest or oocyte elimination by apoptosis (116). Of note, short-term resveratrol treatment improves age-related infertility coincided with increased ovarian expression of sirtuins including SIRT7 (139). Treatment with the herbal extract celastrol as well as the endogenous hormone melatonin protected ovarian granulosa cells from oxidative stress and increased their survival by upregulating SIRT7 and other sirtuins (140). Together, SIRT7 expression in oocytes is indispensable for preventing age-related decline in fertility.

### 5.6. Cancer

Most cancer types show age-related incidence trajectories. A biological connection between cancer and aging exists as well, as both anomalies share common hallmarks such as genomic instability, telomere attrition, epigenetic alterations, abnormal proteostasis, altered cellular metabolism, and heightened inflammation (141). Mounting evidence supports that loss of *SIRT7* promotes cancer metastasis (96, 142), resistance to chemotherapy (143, 144), and tumor survival under energetic stress (52, 144). SIRT7 is declined in chemoresistant breast cancer cells; this inhibits insulin uptake, possibly due to reduced expression of the insulin receptor and substrate (145). It also deacetylates FKBP prolyl isomerase 1 (FKBP1), which dephosphorylates the tumor-promoting AKT and suppresses downstream signaling. This mechanism sensitizes breast cancer cells to the chemotherapeutic agents paclitaxel and epirubicin (144). Moreover, stabilization of SIRT7 by AMPK/glycogen synthase kinase 3 beta (GSK3β)-mediated sequential phosphorylation elicits antitumor effects by inhibiting S-phase kinase associated protein 2 (SKP2)-mediated K63-linked AKT activation. Prolonged intermittent fasting (IF) or chemotherapy inhibits the GSK3β-SIRT7 axis via ERK2, whereas treatment with the MAPK kinase (MEK)/ERK inhibitor U1206, combined with chemotherapy (doxorubicin) and IF, enhances the antitumor effects (94). SIRT7 downregulation promotes breast cancer metastasis by increasing TGF-β signaling; this effect can be reversed by resveratrol treatment, which activates SIRT7 and promotes deacetylation of SMAD4, a central mediator of TGF-β signaling, thereby increasing cancer patient survival (96). As a feedback loop, HDAC8 and SMAD3/4 heterotrimer complex suppresses *SIRT7* transcription through chromatin remodeling and thus relieves SMAD4 from SIRT7-mediated inhibition. This action causes TGF-β signaling hyperactivation in metastatic breast cancer cells (143). In another scenario, SIRT7 interacts with EST-1 transcription factor and subsequently deacetylates histone at the TEK promoter, thereby inhibiting breast cancer metastasis (146). SIRT7 reduction also promotes progression of malignant breast cancer by inhibiting isocitrate dehydrogenase 1 (IDH1) gene transcription. Mechanistically, inhibition of IDH1 reduces α-ketoglutarate (α-KG) synthesis, causing epigenetic and metabolic reprograming and thus promoting proliferation of breast cancer cells (147).

**FIGURE 12.**
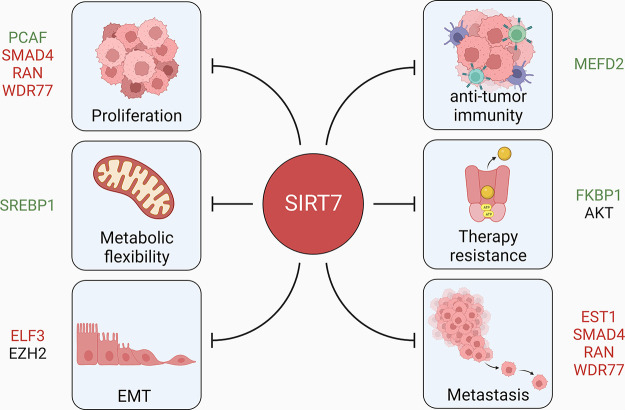
Tumor-suppressive functions of SIRT7. SIRT7 counteracts cancer by inhibiting key events involved in cancer progression including proliferation, metabolic flexibility, epithelial-to-mesenchymal transition (EMT), metastasis, therapy resistance, and antitumor immunity in different cancer types. Molecular targets of SIRT7 are shown in color: red, downregulated; green, upregulated; black, indirectly downregulated.

In colorectal cancer, SIRT7 works downstream of the gene associated with retinoic and interferon-induced mortality 19 (GRIM-19) and triggers PCAF-mediated MDM2 ubiquitination and degradation, thereby stabilizing p53 and inhibiting cell proliferation (148). Alternatively, SIRT7 deacetylates RAS-related nuclear protein (RAN), an event that promotes nuclear accumulation of STAT3 and subsequent autophagic response, inhibiting proliferation and metastasis of colorectal cancer cells. This axis is turned in favor of cancer cells by ribosomal L1 domain containing 1 (RSL1D1), which competitively binds to SIRT7 and inhibits RAN deacetylation (149). SIRT7 also deacetylates WD repeat domain 77 (WDR77) and thereby disrupts the interaction between WDR77 and protein arginine methyltransferase 5 (PRMT5), protecting against proliferation and migration of colon cancer cells (150). SIRT7 level is also lower in advanced-stage muscle-invasive tumors than in papillary bladder cancer (151). Downregulation of SIRT7 orchestrates EMT and promotes metastasis in bladder cancer cells because of the inhibition of E-cadherin mediated by enhancer of zeste homolog 2 (EZH2) methyltransferase (151). Later studies report that SIRT7 has similar roles in oral squamous cell carcinoma (OSCC): it is successively downregulated in OSCC and inhibits EMT in vitro and lung metastasis in vivo (142); miR-770 promotes OSCC metastasis by targeting SIRT7 and inhibiting the downregulation of SMAD4 signaling (152). In esophageal squamous cell carcinoma (ESCC), SIRT7 deacetylates the promoter of E74 like ETS transcription factor 3 (ELF3), rendering it no longer able to suppress miR-144-3p, a posttranscriptional suppressor of zinc finger E-box binding homeobox 1/2 (ZEB1/2) EMT markers. This SIRT7/ELF3/miR-144-3p axis is fine-tuned by Linc00886, which interacts with and recruits SIRT7 to ELF3 promoter in the first place (153). In hepatocellular carcinoma (HCC), the elevated programmed cell death ligand 1 (PD-L1) expression downstream of myocyte enhancer factor 2 (MEF2D) orchestrates immune escape. SIRT7 deacetylates MEF2D to inhibit PD-L1, thereby promoting antitumor immunity in the HCC cells (154). Finally, SIRT7 level may serve as a biomarker to monitor treatment response in acute myeloid leukemia (AML) or chronic myelogenous leukemia (CML) patients, as it is restored to normal level upon a positive treatment response but is reduced during disease relapse (30). Collectively, these tumor-inhibitory roles attributed to SIRT7 are mainly tissue specific and context dependent and are of a secondary nature, which warrants more detailed investigation before establishing SIRT7 as a key molecular target in cancer (FIGURE 12).

## 6. SIRT7-BASED INTERVENTIONS DECELERATE AGING

Progressive decline in SIRT levels and activity is evident in aging (155). Resveratrol, a polyphenol enriched in berries and red wine, was initially identified as an activator of SIRT1 (156, 157) but was later shown to directly activate SIRT7 (25, 96). Resveratrol treatment improves the health of aging rodents (158–160). It elongates life span in silkworms by relieving oxidative stress through activation of the SIRT7-FoxO-GST axis (161). Recently, Sun et al. (88) confirmed that AAV-mediated *SIRT7* gene delivery can ameliorate aging features and extends life span in a progeria mouse model; this opens an avenue for gene therapy in aging intervention.

NAD^+^ is regularly recycled during energy metabolism and ATP production but also works as a substrate for a plethora of cellular enzymes, including SIRTs, to maintain cellular homeostasis. NAD^+^ levels decline in physiologically aged mice and premature aging models (35, 36), and such NAD^+^ insufficiency is sensed through multiple cellular mechanisms that involve SIRTs (162). NAD^+^ precursor supplementation incorporates a wide range of benefits such as improving glucose homeostasis (163), maintaining genomic integrity (35), restoring muscle mass (164), and preventing heart failure (165), steatosis (166), and glaucoma (167) in aged mice. Pharmacological inhibition of alpha-amino-beta-carboxy-muconate-semialdehyde decarboxylase (ACMSD), a key modulator of cellular NAD^+^ synthesis, improves mitochondrial homeostasis and healthiness in nematodes and mice (168). Transfer of extracellular vesicles containing extracellular nicotinamide phosphoribosyltransferase (eNAMPT, a rate-limiting synthase in NAD^+^ salvage) from young to aged mice extends life span (169). Food supplementation with tryptophan, vitamin B_3_ (nicotinamide, NAM), and NAD^+^ intermediates such as nicotinamide mononucleotide (NMN), nicotinamide riboside (NR), and nicotinic acid riboside (NaR) stimulates NAD^+^ production (170). NMN treatment increases the life span of BubR1 (a mitotic checkpoint kinase)-null mice, a model of premature aging (171). NR supplementation rejuvenates intestinal stem cells in old mice and increases life span (∼5%) (172, 173). NR administration improves health in a mouse model of premature aging caused by deficient DNA repair, *Atm*^–/–^ mice (174). Of note, NR is approved for clinical use, is proven orally available and safe, and is able to increase blood NAD^+^ metabolism in humans (175).

Changing eating habits has great potential to attenuate the aging process. Both CR and IF are proven effective health-promoting and life span-extending dietary manipulations. CR induces SIRT7 auto-ADP-ribosylation, which then interacts with mH2A1 in mouse liver to modulate autophagy response and aging (80). This implies that impaired autophagy due to an abrogated SIRT7-mH2A1 axis might be the culprit behind the accelerated aging observed in *Sirt7*-KO mice. CR also helps prevent SIRT7 decline over the course of aging in organs such as liver (80). IF or mild energetic stress upregulates SIRT7 via the sequential phosphorylation mediated by AMPK and GSK3β and thus inhibits proteasome degradation (94). Further studies are needed to determine whether this effect also underlies the antiaging benefit of CR. Intriguingly, short-term CR and refeeding upregulate SIRT7 expression in the cardiac tissue of old rats but not in the liver of both young and old rats (29). An independent study found that *SIRT4* and *SIRT7* expression decreased in the liver of C57BL/6 mice when the degree of CR increased (0% to 40% CR) over 3 mo, probably because rDNA transcription was shut down in liver tissues to conserve energy (176). Of particular note, the long-term effect of severe nutritional intervention on SIRT7 might be opposite (94). In addition, CR, IF, and physical activity can enhance the cellular NAD^+^ pool, which aids in improving SIRT’s enzymatic activity. Overall, nutritional and pharmacological interventions may slow down the aging process partly by alleviating the decline in SIRT7 activity, although knowledge of the in-depth molecular mechanisms is still evolving.

## 7. CONFLICTING EVIDENCE ON SIRT7’S ROLE IN AGING, RELATED PATHOLOGIES, AND LONGEVITY

Although disease-associated faster aging and shortened life span are apparent in *Sirt7*-KO mice (with exon 4–9 deletion) (25), and independent reports have affirmed that these animals experience hepatic (82, 83) and cardiac anomalies (25, 83) as well as WAT reduction and weight loss (82, 106, 112), conflicting findings have also been reported. Yoshizawa et al. found that *Sirt7*-KO mice are resistant to high-fat diet (HFD)-induced fatty liver disease and accumulate lower levels of triglycerides in the liver. At the molecular level, SIRT7 stabilizes TGF-β-activated kinase 1 (TAK1) by inhibiting the E3 ubiquitin ligase complex of damage-specific DNA binding protein 1 (DDB1)/cullin 4B (CUL4B)/DDB1-CUL4-associated factor 1 (DCAF1), thus promoting fatty acid uptake, lipid synthesis, and storage. SIRT7 deacetylates DDB1, which prevents its binding to DCAF1, and reduced TAK1 levels protect against hepatosteatosis in *Sirt7*-KO mice (112, 177). Mizumoto et al. (178) reported that these *Sirt7*-KO mice did not experience any cardiac fibrosis or dysfunction and observed extended life span in male mice (but not in female), which they attributed to protection against glucose and insulin intolerance. Ryu et al. (83)generated an independent mouse line with deletion of exons 6–9, and these *Sirt7*-KO mice experienced age-related anomalies such as hepatosteatosis, cardiac dysfunction, loss of hearing, and reduced exercise performance although little change in body weight and composition was observed. The differences in genetic background, construction strategy, and experimental and housing conditions of *Sirt7*-KO mice are plausible explanations for the seemingly conflicting roles of SIRT7 in relation to metabolic homeostasis, aging, and longevity. This warrants further exploration before moving toward clinical investigations.

Discrepancies have also been observed in the context of SIRT7’s role in aging-related pathologies. A key example is Alzheimer’s disease (AD), a neurodegenerative disease stemming from amyloid-β (Aβ) accumulation in brain that leads to cognitive decline and irreversible memory loss. Elevated SIRT7 expression is observed in the cortex region of brain in AD patients (179). Interestingly, this is somewhat in line with changes observed in SIRT7 expression in the frontal lobe of brain during normal aging (180). Elevated SIRT7 promotes Aβ aggregation by upregulating NADPH oxidase 4 (NOX4) expression, which leads to oxidative stress and apoptosis in neuronal cells, whereas *SIRT7* knockdown rescues cells from apoptotic death (179), although in vivo validation is warranted. During pulmonary arterial hypertension (PAH) pathogenesis, SIRT7 upregulates AKT in a JNK-dependent manner that, in turn, activates lipogenic enzymes ATP-citrate lyase and acetyl-CoA carboxylase, promotes lipid deposition, and remodels the pulmonary vasculature in favor of PAH (181). An independent study found that *Sirt7*-KO mice are resistant to aging- and forced exercise-induced osteoarthritis. Increased SIRT7 in cartilage-depositing chondrocytes inhibited the transcriptional activity of SRY-box transcription factor 9 (SOX9), thereby reducing extracellular matrix (ECM) deposition and disrupting cartilage homeostasis (115).

Diabetes, characterized simply as hyperglycemia and/or acute-phase glucose intolerance, can arise as a consequence of insulin deficiency due to loss of pancreatic β-cells (type 1 diabetes mellitus) or impaired insulin secretion and insulin resistance (type 2 diabetes mellitus). Age and obesity are major risk factors for diabetes (182). SIRT7 level is higher in subcutaneous and visceral adipose tissues of obese individuals than in those of healthy individuals (183). When *Sirt7*-KO mice are fed a high-fat diet, they gain weight more slowly and accumulate less epididymal WAT than control mice. The mice have lower insulin and fasting blood glucose levels and better tolerate insulin and glucose (112). Aged *Sirt7*-KO mice express high levels of hepatic activating transcription factor 4 (*Atf4*) compared to control mice, which helps sustain glucose and insulin intolerance by maintaining high serum levels of hepatokine fibroblast growth factor 21 (FGF21) in old age (178). In agreement with this, liver-specific *Lsd1*-KO mice, which have reduced SIRT7 activity, are protected from glucose intolerance, partly attributable to the induction of FGF21 (84).

The roles of SIRT7 in cancer are highly context dependent and have been equally reported to be tumor suppressive and oncogenic. The earliest evidence linking SIRT7 to cancer noted that SIRT7 level is higher in breast tumors than in normal breast tissue and is positively associated with a higher incidence of node-positive breast cancer (184). SIRT7 has since been linked to advanced tumor stages and high-histological grade tumors that metastasize to lymph nodes and distant sites and to worse prognosis and poor survival in breast (185, 186), liver (58, 187, 188), colorectal (189), pancreatic (191) and prostate (192, 193) cancers.

Elevated SIRT7 expression in certain diseased tissue may not reflect and be in line with age-related changes in the rest of the body. In such scenarios, the priority of targeting aging may become secondary to targeting the disease. Therefore, efforts are being made to develop potent inhibitors of SIRT7 as well. For instance, a chemical screening identified ID:97491 as a potent SIRT7 inhibitor, which induces p53-mediated apoptotic response in vitro and inhibits xenograft tumor growth (194). Two cyclic tripeptides, one with a thiourea-type catalytic inhibitory mechanism and the other with a carboxamide-type catalytic inhibitory mechanism, have been reported as inhibitors of SIRT7 deacetylase; however, no data were presented for their inhibitory role on SIRT7 activity in a biological system (195). Two small molecules, namely 2800Z and 40569Z, are also proposed to actively inhibit SIRT7 deacetylation activity and sensitize HCC cells to sorafenib treatment both in vitro and in vivo (196). Furthermore, two naphthoquinones, namely menadione and plumbagin, were recently reported to inhibit deacetylase activity of SIRT7 and thus hamper DNA repair, rDNA transcription, and pre-rRNA processing (197). Identification of potent SIRT7 inhibitors is still in early stages, and it will take time to explore their potential in clinical settings before approval for human use.

## 8. SIRT7: MOLECULAR GUARD OF HEALTH AND LONGEVITY

The hallmarks of aging include causes of age-related damage, responses to damage, and drivers of age-related phenotypes (2). These hallmarks are devised based on the criteria that they exhibit age-related manifestation; their experimental perturbation accelerates aging; and they provide opportunity for therapeutic interventions that decelerate, stop, or reverse the aging process (198). Here we demonstrate that SIRT7 represents an intrinsic molecular defense that epiregulates the genome via histone modification, the transcriptome via RNA posttranscriptional modification, and the proteome via posttranslational modification against multiple stresses and aging, whereas SIRT7 expression and activity decline with aging; experimental perturbations of SIRT7 disrupt metabolic homeostasis and accelerate systemic aging; and SIRT7 protects against aging-related pathologies and SIRT7-based interventions decelerate aging. Thus, we present SIRT7 as a key in orchestrating adaptive stress responses and safeguarding against aging, which undoubtedly affirms SIRT7 as a molecular guard of health and longevity.

Although pharmacological targeting of aging is still in its infancy, research has opened a gateway for the scientific community to explore small-molecule regulators of health and longevity. SIRTs are undoubtedly the most targetable molecules to counteract the aging process. Indeed, specific activators of SIRTs are being developed; e.g., SRT1720 is a SIRT1 activator, more potent than resveratrol, and extends life span and improves health span in mice (199–201). MDL-800 was discovered as a first-in-class small-molecule activator of cellular SIRT6 (202). It improves the genomic stability and pluripotency of aged induced pluripotent stem cells (iPSCs) by activating the DNA repair machinery (203), alleviates liver fibrosis by increasing SMAD2 deacetylation (204), and inhibits cell proliferation in multiple cancer types including HCC (202) and non-small cell lung cancer (NSCLC) (205). There is a need to identify potent and specific activators of SIRT7 since such molecules hold great potential to quickly move from bench to bedside as antiaging therapeutics.

## 9. OUTSTANDING QUESTIONS

- What are the precise molecular mechanisms by which SIRT7 maintains cellular homeostasis? Although multiple enzymatic activities have been attributed to SIRT7, the major focus remains on its deacetylation activity so far. Other enzymatic and enzyme-independent activities of SIRT7 need to be explored.
- What is the biological relevance of SIRT7-mediated deacetylation of RNA ac4C modification? What else is on the list of SIRT7 targets?
- How do SIRTs interact with each other? Do SIRTs compete for substrates? Although a few context-dependent interactions between SIRTs have been established, further investigation is needed to elucidate the synergistic effects of SIRTs in different physiological and pathological contexts.
- What are the most promising approaches for SIRT7 manipulation in specific tissues? In this context, adeno-associated virus (AAV) systems, exosomes, and other mRNA direct delivery techniques may come into play in addition to conventional administration of activators to boost the progress of use of SIRTs in medicine.
- Would restoring SIRT7 to target aging and age-related pathologies be safe in clinical settings? Although preclinical findings support the notion that restoring SIRT7 slows the aging process and ensures healthy aging and longevity, it is necessary to evaluate the potential risks and side effects.

## GRANTS

The present study was supported by grants from the National Natural Science Foundation of China (grant nos. 82125012, 92249304, and 82061160495), the National Key R&D Program of China (grant no. 2021ZD0202400), the Guangdong Basic and Applied Basic Research Foundation (grant no. 2021B1515120062), and the Shenzhen Municipal Commission of Science and Technology Innovation (grant nos. JCYJ20220818100016035 and JCYJ20220818100009020).

## DISCLOSURES

No conflicts of interest, financial or otherwise, are declared by the authors.

## AUTHOR CONTRIBUTIONS

U.R. prepared figures; U.R. drafted manuscript; U.R., X.T., Z.L., and B.L. edited and revised manuscript; U.R., X.T., Z.L., and B.L. approved final version of manuscript.
